# Compensation Method for Pipeline Centerline Measurement of in-Line Inspection during Odometer Slips Based on Multi-Sensor Fusion and LSTM Network

**DOI:** 10.3390/s19173740

**Published:** 2019-08-29

**Authors:** Shucong Liu, Dezhi Zheng, Rui Li

**Affiliations:** 1 School of Instrumentation and Optoelectronic Engineering, Beihang University, Beijing 100191, China; 2 Institute of Disaster Prevention, Sanhe 065201, China; 3 School of Automation Science and Electrical Engineering, Beihang University, Beijing 100191, China

**Keywords:** pipeline centerline measurement, in-line inspection, multi-sensor fusion, inertial measurement unit, LSTM

## Abstract

The accurate measurement of pipeline centerline coordinates is of great significance to the management of oil and gas pipelines and energy transportation security. The main method for pipeline centerline measurement is in-line inspection technology based on multi-sensor data fusion, which combines the inertial measurement unit (IMU), above-ground marker, and odometer. However, the observation of velocity is not accurate because the odometer often slips in the actual inspection, which greatly affects the accuracy of centerline measurement. In this paper, we propose a new compensation method for oil and gas pipeline centerline measurement based on a long short-term memory (LSTM) network during the occurrence of odometer slip. The field test results indicated that the mean of absolute position errors reduced from 8.75 to 2.02 m. The proposed method could effectively reduce the errors and improve the accuracy of pipeline centerline measurement during odometer slips.

## 1. Introduction

Pipeline transportation is the most economical and reasonable method for transporting oil and gas because of its low transportation cost, stable continuous supply, and large transportation capacity [1,2]. With the increase in pipeline quantity and pipeline age, pipeline leakage and deflagration accidents caused by corrosion, accidental damage, and other reasons are becoming increasingly serious, which causes great damage to life and property and safety of people and the ecological environment [3,4]. In order to ensure safe transportation of the pipeline, it is necessary to carry out pipeline inspections. The internationally recognized conventional pipeline inspection method is pipeline in-line inspection (ILI), the ILI tools can be mounted using different sensors for different parameter measurements. In order to maintain the pipeline safety and reduce accidents due to pipeline displacement and deformation [1,5], the pipeline centerline and coordinate measurement system has been playing a big role in positioning and safety [6].

The common practice is to use in-line inspection [7] based on strapdown inertial navigation technology to realize the pipeline centerline and coordinate measurement [8,9]. At present, the geographic trajectory of underground pipelines is mainly measured by high-precision inertial measurement units (IMUs) and micro-electromechanical-systems (MEMS) inertial measurement units (MEMS-IMUs) [10,11]. Inertial measurement units can sense the angular velocity and specific force of the pipeline inspector in three directions, and by navigation calculation the position, velocity, and attitude navigation information can be obtained. Due to the device error influence of the IMU itself, the position, velocity, and attitude errors of the centerline in-line inspector system will accumulate rapidly, which will seriously affect the measurement accuracy. Thus, it is necessary to compensate for these errors by combining an odometer, an above-ground marker (AGM), and other observation information.

An odometer can measure forward distance of the pipeline centerline in-line inspector, and the distance can be converted to forward speed to suppress the accumulation of navigation and positioning errors. An above-ground marker whose coordinates are known provides position correction information for pipeline centerline in-line inspectors. The navigation calculation combining IMU with AGM and odometer can significantly improve the positioning accuracy. However, during the actual inspection, owing to the slip of the odometer (especially in the bend), the odometer cannot guarantee the continuous and stable output of the speed information. When the odometer slips, the IMU error diverges rapidly without restraint. Hence, it is necessary to introduce new methods for pipeline navigation to increase the positioning accuracy. Recently, various algorithms and approaches have been proposed to compensate the positioning errors due to slip and slide. In [12], multi-sensor data fusion was adopted using adaptive fuzzy Kalman filtering for the errors to not only detect any occurrence of slip and slide in the system, but also compensate the resulting errors. Some scholars proposed many practical intelligent methods based on artificial intelligence (AI) for inertial navigation error suppression [13,14,15,16,17,18,19]. However, currently no relevant research is found in the error compensation of pipeline centerline measurement during the occurrence of odometer slip.

With the aim to increase the position accuracy for small-diameter pipelines, a compensation method was proposed to correct velocity errors due to odometer slip and improve the measurement accuracy. The actual measurement results show that this method can be used to effectively restrain the inertial navigation error divergence and improve the positioning accuracy of the pipeline centerline measurement. Firstly, the coordinates of the pipeline centerline were obtained by an extended Kalman filter (EKF) using the IMU, above-ground marker, and odometer. Secondly, the acceleration information processed using the wavelet transform (WT) was used to identify every girth weld. According to the girth weld, the measured length of each section of the pipeline and bend was determined. Then the measured length was compared with the engineering data to determine whether the odometer slipped. If the errors are in the allowable range, the slip can be ignored, and the velocity data of the odometer can be used as the training set for the model. If the errors exceed the allowable range, the slip of the odometer cannot be ignored, and the odometer velocity needs to be corrected. Finally, the model based on long short-term memory (LSTM) network and the odometer velocity when the odometer works was trained to predict the velocity error when the odometer slipped, and the predicted velocity error could be used for coordinate calculation and centerline measurement.

## 2. Principle of IMU in-Line Inspection Measurement

**Figure 1:**
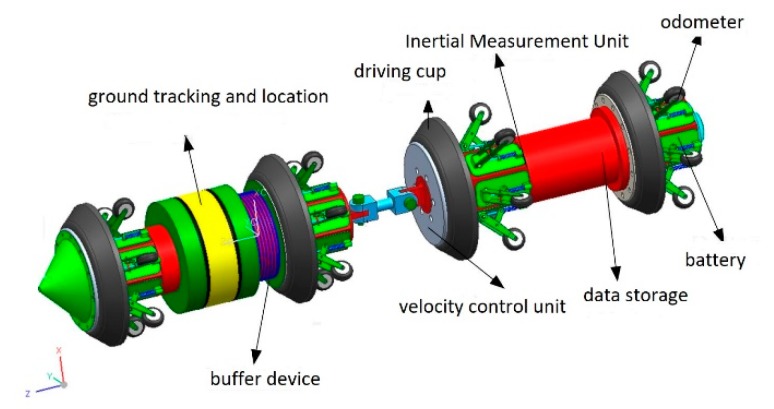
Pipeline centerline measurement system.

The IMU in-line inspection measurement system is pushed forward by the oil and gas inside the buried pipeline. The IMU is used to measure the angle, orientation, and distance of the system in real time. When the initial position is known, the geographic coordinates of the inspection system at each sampling time can be calculated in order to achieve positioning. Because of the errors of the gyroscope and accelerometer in the inertial device, the errors of the sensor are amplified with time after integration. Other sensors, such as the odometer and ground tracking system, are used to correct the errors in order to achieve accurate positioning. The pipeline centerline measurement system based on IMU, shown in Figure 1, is composed of MEMS-IMU, odometer, ground tracking system, velocity control unit, data storage unit, and so on.

**Figure 2:**
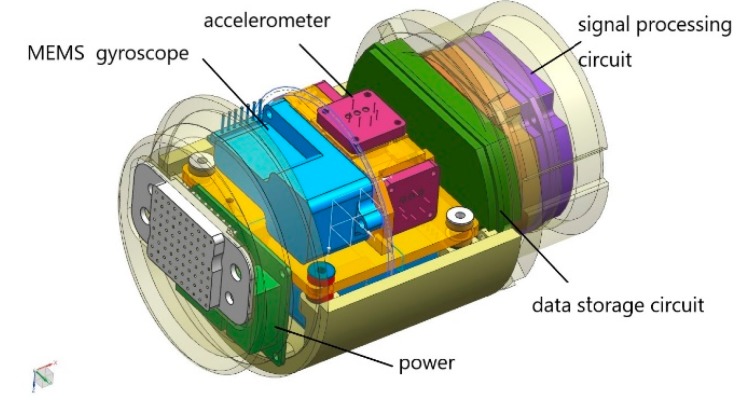
Inertial measurement unit components. MEMS—micro-electromechanical system.

The IMU is mainly composed of three TS-02X1 MEMS gyroscopes, three JL-01FX1 accelerometers, HJ-148A power supply module, FG-167B integrated information processing circuit, FG-144C data storage circuit, information processing software, and so on, as shown in Figure 2. The MEMS gyroscope senses the angular motion of the carrier in three axes, to measure the angular velocity of the carrier, and the analog signal proportional to the angular velocity of the carrier is output. The accelerometer senses the linear motion of the carrier in three axes to measure the linear acceleration of the carrier and output frequency signals proportional to the acceleration. The signal processing circuit collects the outputs of angular rate signals of gyro, the frequency signals of accelerometer, temperature signals, and the frequency signals of the odometer. The data storage circuit saves the collected data in real time. The data stored in the inertial measurement device can been obtained to carry out navigation calculation, and odometer information and ground marking information are used to correct navigation errors, so as to obtain position, attitude angle, and speed information. The process of navigation calculation can be found in some references [20].

The IMU is the main sensor for collecting pipeline in-line inspection gauge (PIG) motion data. The simplified linear error equations that describe the dynamic behavior of the inertial measurement unit are given by

(1) $$\left\{ \begin{array}{c} \delta {\dot{v}}^{n}=f^{n}\times \Psi +C_{b}^{n}\delta f^{b}-\left(2\omega_{ie}^{n}+\omega_{en}^{n}\right)\times \delta v^{n}\\ -\left(2\delta \omega_{ie}^{n}+\delta \omega_{en}^{n}\right)\times v^{n}+\delta g_{l}\\ \dot{\Psi }=-\omega_{in}^{n}\times \Psi +\delta \omega_{in}^{n}-C_{b}^{n}\delta \omega_{ib}^{b}\\ \delta \dot{L}=\frac{1}{\left(R_{N}+h\right)}\delta v_{N}-\frac{v_{N}}{{\left(R_{N}+h\right)}^{2}}\delta h\\ \delta \dot{l}=\frac{1}{\left(R_{E}+h\right)cosL}\delta v_{E}+\frac{v_{E}sinL}{\left(R_{E}+h\right)cos^{2}L}\delta L\\ -\frac{v_{E}}{{\left(R_{E}+h\right)}^{2}cosL}\delta h\\ \delta \dot{h}=-\delta v_{D} \end{array},\right.$$

where $\delta v^{n}$ is velocity error; Ψ is attitude error; *L* and *l* are, respectively, latitude and longitude; and *h* is the elevation. $R_{E}$ and $R_{N}$ are the Earth radius under the WGS-84 geography coordinator system. $f^{n}$ is specific force value, $C_{b}^{n}$ is the transmit matrix from the PIG carrier coordinate system into the local navigation coordinate system, $\omega_{ie}^{n}$ is the Earth angular velocity in the navigation coordinate system, $\omega_{en}^{n}$ is the angular rate relative to the Earth navigation coordinate system. $v_{N}$, $v_{E},\;\mathrm{and}\;v_{D}$ are velocity in north, east, and down direction, respectively [20]. The centerline measurement system based on the MEMS-IMU is a non-linear continuous system, and the standard Kalman filter is unsuitable for the state or parameter estimation of the system. Therefore, the EKF can be used to linearize the error state of the nonlinear continuous system locally to achieve an optimal estimation. The dynamic measurement model formed in the previous section is used to estimate the system state. The estimated state vectors include the position error, $\delta r^{n}$; velocity error, $\delta v^{n}$; attitude error, Ψ; gyroscope random bias error, $\delta b_{g};$ and acceleration error, $\delta b_{a}$, total fifteen dimensions, as follows [20]:

(2) $$\delta x={[\begin{array}{ccccc} \delta r^{n} & \delta v^{n} & \Psi & \delta b_{g} & \delta b_{a} \end{array}]}^{Τ},$$

$\mathrm{where}\;\delta r^{n}=\left[\begin{array}{ccc} \delta L & \delta l & \delta h \end{array}\right]$ are north position error, east position error, and vertical position error in the navigation coordinate system. $\delta v^{n}=\left[\begin{array}{ccc} \delta v_{n} & \delta v_{E} & \delta v_{D} \end{array}\right]$ are north, east, and vertical velocity error in the navigation coordinate system. $\Psi =\left[\begin{array}{ccc} \alpha & \beta & \gamma \end{array}\right]$ are roll angle error, pitch angle error, and heading angle error. The error model of IMU can be represented by a continuous linear stochastic system as follows:

(3) $$\delta \dot{x}\left(t\right)=F\left(t\right)\delta x\left(t\right)+G\left(t\right)\omega \left(t\right).$$

In the formula, F(t) is the dynamic matrix, δx(t) is the state vector, G(t) is the noise input coefficient matrix, and ω(t) is the noise vector. The discretized error state model of the inertial navigation system can be expressed as follows:

(4) $$\delta x_{k+1}=\Phi_{k}\delta x_{k}+G_{k}\omega_{k},$$

$\mathrm{where}\;\delta x_{k+1}$ is a 15×1 order state vector, $\Phi_{k}$ is a 15×15 order transformation matrix, $G_{k}$ is a 15×1 noise distribution matrix, and $\omega_{k}$ is Gauss white noise with unit variance. By applying the Taylor series expansion and ignoring the higher-order terms, the linear system model can be expressed as follows:

(5) $$\delta x_{k+1}=\left[\begin{array}{ccccc} I & F_{1} & 0 & 0 & 0\\ 0 & I & F_{2} & 0 & F_{3}\\ 0 & F_{4} & I & F_{3} & 0\\ 0 & 0 & 0 & F_{5} & 0\\ 0 & 0 & 0 & 0 & F_{6} \end{array}\right]\left[\begin{array}{c} \delta r_{k}\\ \delta v_{k}\\ \delta \varepsilon \\ \delta b_{g}\\ \delta b_{a} \end{array}\right]+\left[\begin{array}{c} \sigma_{r}\\ \sigma_{v}\\ \sigma_{\varepsilon }\\ F_{7}\\ F_{8} \end{array}\right]\omega_{k},$$

(6) $$\begin{array}{c} \delta r_{k}={\left[\begin{array}{ccc} \delta L_{k} & \delta l_{k} & \delta h_{k} \end{array}\right]}^{Τ},\delta v_{k}={\left[\begin{array}{ccc} \delta v_{k}^{N} & \delta v_{k}^{E} & \delta v_{k}^{D} \end{array}\right]}^{Τ}\\ \delta \varepsilon_{k}={\left[\begin{array}{ccc} \delta \alpha_{k} & \delta \beta_{k} & \delta r_{k} \end{array}\right]}^{Τ},\delta b_{g}={\left[\begin{array}{ccc} \delta b_{gx} & \delta b_{gy} & \delta b_{gz} \end{array}\right]}^{Τ}\\ \delta b_{a}={\left[\begin{array}{ccc} \delta b_{ax} & \delta b_{ay} & \delta b_{az} \end{array}\right]}^{Τ} \end{array},$$

where $\sigma_{r},\;\sigma_{v},\;\mathrm{and}\;\sigma_{\varepsilon }\;\mathrm{are}$ white noise of position, velocity, and angle for IMU. The estimation error of EKF is the latest time estimate of the system state vector, and the filter works in a closed loop with an error feedback correction. Therefore, the system error state vector completes the observation update, it is used to correct the navigation state and parameters and reset the error state vector to zero. The state updating equation is expressed in the discrete form as follows:

(7) $$\delta {\dot{x}}_{k}=K_{k}\left(Z_{k}-H{\hat{x}}_{k|k-1}\right),$$

where $K_{k}$ represents the filter gain matrix. *H* is the design matrix. The corresponding covariance matrix is:

(8) $$P_{k|k-1}=\Phi_{k,k-1}P_{k-1}\Phi_{k,k-1}^{Τ}+Q_{k}P_{k|k-1}.$$

In the formula, $P_{k-1}$ is the covariance matrix for the error state vector, $\Phi_{k,k-1}$ is the state transition matrix, and $Q_{k}$ is the covariance matrix of the system noise. The EKF updates the covariance matrix of the estimated state error vector as follows:

(9) $$K_{k}=P_{k|k-1}H_{k}^{Τ}{\left(H_{k}P_{k|k-1}+R_{k}\right)}^{-1},$$

(10) $$P_{k}=\left(I-K_{k}H_{k}\right)P_{k|k-1}{\left(I-K_{k}H_{k}\right)}^{Τ}+K_{k}R_{k}K_{k}^{Τ},$$

$\mathrm{where}\;R_{k}$ is the covariance matrix for the observation vectors. Further details regarding the EKF can be found in other references [21,22,23].

**Figure 3:**
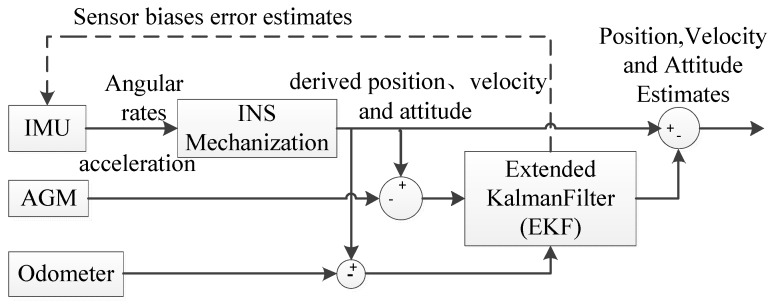
Centerline calculation based on multi-sensor data fusion algorithm. IMU—inertial measurement unit; INS—inertial navigation system; AGM—above-ground marker.

The principle of the pipeline centerline measurement and calculation based on MEMS-IMU with the adopted multi-sensor data fusion algorithm based on EKF is shown in Figure 3. The IMU provides the acceleration and angular velocity, and the AGM and odometer provide the position and velocity information as a reference for correcting the IMU errors. An EKF is used to fuse the measurements from the IMU, odometer, and reference markers.

In centerline measurement and calculation, positive EKF filtering, interval smoothing filtering, and output correction are firstly adopted. Then data are counted from the end and a reverse filtering correction is carried out, finally the results of the two are fused and corrected to improve the measurement accuracy. After the above post-processing and calculation, the geographic coordinates of the pipeline centerline in-line inspector can be obtained, thus, the trajectory of the pipeline centerline can also be determined.

## 3. Description of Compensation Method

### 3.1. Description of Error Compensation Method

**Figure 4:**
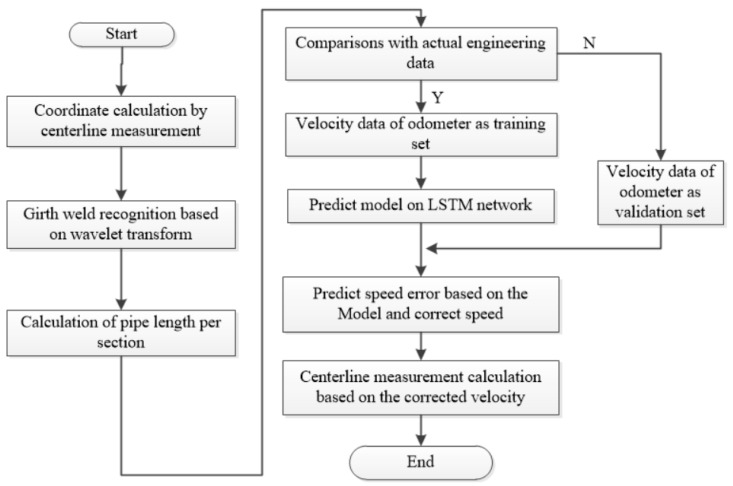
Flow chart of the compensation method. LSTM—long short-term memory.

The odometer may slip at the girth weld when the pipeline centerline measurement system travels along the pipeline, thus resulting in inaccurate velocity information. As the velocity information at the slip of the girth weld cannot be used for correction, it is necessary to identify and estimate the velocity error that cannot work normally. According to the velocity error of the odometer when it works normally, the method-based AI can be used to build the model to predict the velocity error when it works abnormally. The specific flow chart is shown in Figure 4. Firstly, the geographic coordinates of the pipeline centerline are obtained through centerline calculation using the IMU, AGM, and odometer. Secondly, it is necessary to identify the girth weld with acceleration based on the wavelet transform, and to identify the measured length of each pipe and bend according to the girth weld. Then the length is compared with the engineering data. If they are consistent, the odometer velocity is regarded as the training set, and if not, the odometer velocity is required to be predicted and corrected. Finally, the velocity error when the odometer slips can be corrected, and the corrected velocity can be used for coordinate calculation and centerline measurement.

The coordinate calculation process of the pipeline centerline is detailed in Figure 3. Next sections are respectively girth weld identification and prediction model on LSTM network.

### 3.2. Girth Weld Identification of Pipeline Based on Wavelet Transformation

In general, a long-distance oil and gas transportation pipeline is constructed by connecting straight pipelines and other fittings (elbows, T fittings, and valves) [24]. During the construction on site, in general, the straight pipelines are connected with other fittings using girth welds or flanges. In the pipeline construction, the welded joints are firm and durable, the strength of joints is greater, and the tightness of connection is higher, such that the weld connection is a universal and important means of connection for pipelines [25]. The connection welds are called girth welds. The odometer slips easily at the weld, and, thus, it is necessary to identify the weld.

When the centerline in-line inspection system is running in the pipeline, the accelerometer of the inertial measurement unit is sensitive to any impulse and vibration against the in-line inspection system in the pipeline. When the in-line inspection system passes each girth weld, three orthogonal mounting accelerometers produce clear pink signals when they are vibrated. If these accelerometers signals are effectively detected, the girth weld information of the entire pipeline may be effectively identified for identifying the odometer slip. Owing to the noises produced by the in-line inspection system in the pipeline, it is difficult to treat the acceleration signals for the identification of the pipeline girth welds. Therefore, before the identification of the girth weld, it is necessary to do effective noise reduction against the acceleration data.

The wavelet transformation [26,27] is useful for eliminating the noises from transient signals and instantaneous signals, and it may restrict any interruption of high-frequency noises and effectively distinguish the high-frequency information from high-frequency noises [28,29]. The wavelet transform can be defined as follows:

(11) $$W_{\psi }f\left[a,b\right]=\frac{1}{\sqrt{\left|a\right|}}\int_{-\infty }^{\infty }\bar{\psi \left(\frac{x-b}{a}\right)}f\left(x\right)dx,$$

(12) $$c_{j,k}=\left[W_{\psi }f\right]\left(2^{-j},k2^{-j}\right).$$

where, $a=2^{-j}$ is called the scale factor, and $b=k2^{-j}$ is the displacement factor, $c_{j,k}$ are the wavelet coefficients.

The actual acceleration signals in the IMU should be low-frequency or steady signals, while the noise signals are high-frequency signals. The orthogonal wavelet db8 is used to perform four-layer decomposition of the acceleration signals, then soft threshold quantization is used to treat the high frequencies of the decomposed signals for de-noising, and finally the low-frequency factors and high-frequency factors after the de-noising of each layer are reconstructed.

In this manner, the signals of each girth weld may be easily identified by use of the acceleration signals after the wavelet de-noising, which provides the basis for further proposal for the mapping of pipelines and the computation of the pipeline length. The measured length of each section of the pipeline is identified according to the girth weld, as compared with the engineering data. If the measured length is consistent with the engineering data, it is regarded as the training set.

### 3.3. Long Short-Term Memory (LSTM)

With the continuous development of deep learning technology, some deep learning models are gradually applied to the study of time series data. A recurrent neural network (RNN) is a kind of neural network with a self-cycling structure, which is very suitable for processing time series data in theory. RNNs are feedforward neural networks with time connection. On the basis of an ordinary multi-layer back propagation (BP) neural network, the RNN adds transverse connections among the hidden layer units. Using a weight matrix, the values of the neurons in the previous time series can be transferred to the current neurons, which allows the neural network to have memory function. The input information of a neuron includes not only the output of the previous layer of nerve cells, but also its own state in the previous channel. It has potential application in dealing with machine learning problems of time series. The disadvantage is that when the original RNN processes long sequences, gradient vanishing will occur. This phenomenon may even lead to a decrease in the accuracy rate as the number of hidden layers increase [30,31].

RNN has produced many variants, in which the LSTM network makes up for the problems of RNN gradient disappearance, gradient explosion, and insufficient long-term memory ability. The LSTM network has been applied in time series related research such as stock prediction, fault time series prediction, speech rsecognition, and so on. LSTM [32,33,34] is an implementation of RNN, which adds memory units to each neuron unit of the hidden layer and combines short-term memory with long-term memory through gate control such that the memory information on the time series can be controlled. Each time the memory information is transmitted between the neurons of the hidden layer, several controllable gates (forgetting gate, input gate, candidate gate, and output gate) are used to control the memory and forgetting degree of the previous and current information, such that the LSTM network has the function of long-term memory. The smart design of the memory cell in the LSTM can effectively solve the problem of gradient vanishing in backpropagation and can be used to learn the input sequence with longer time steps to some extent.

**Figure 5:**
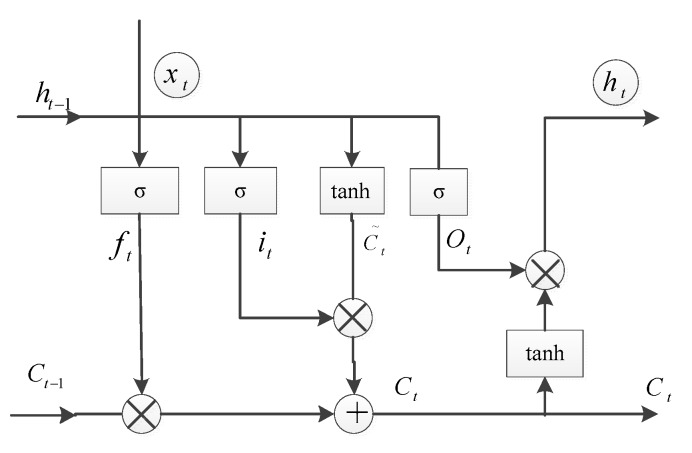
LSTM structure.

Because of the function of long-term memory networks, the LSTM structure is used in the method for predicting the odometer velocity when the odometer slips. The schematic of the LSTM is shown in Figure 5, where $x_{t}$ is a new input, its previous state is $h_{t-1},$ and its previous output is $c_{t-1}$, the cell performs different operations using gates. The specific formula derivation of the LSTM is illustrated in Equations (13)–(18), where σ is a sigmoid function.

The entire process of the LSTM neural network is divided into four steps: The first step is to identify the information that should be forgotten by neurons, which is composed of a sigmoid layer called the forget gate layer. $h_{t-1}$ and $x_{t}$ are the inputs, and each neuron state of $c_{t-1}$ outputs a number between 0 and 1. A “1” indicates that it is completely retained, while a “0” indicates it is completely forgotten.

(13) $$f_{t}=\sigma \left(W_{f}\cdot \left[h_{t-1},x_{t}\right]+b_{f}\right)$$

The second step is to determine the information that is to be stored in neurons. A sigmoid layer called the forget gate layer determines the value update, and then a tanh layer generates a new candidate value, $c_{t}$, which is added to the neuron state.

(14) $$i_{t}=\sigma \left(W_{i}\cdot \left[h_{t-1},x_{t}\right]+b_{i}\right)$$

(15) $${\tilde{C}}_{t}=\tanh\left(W_{c}\cdot \left[h_{t-1},x_{t}\right]+b_{c}\right)$$

The third step is to update the previous state value, $C_{t-1},$ to $C_{t}$, i.e., to multiply the old state by $f_{t}$ to forget the information, and add $i_{t}\cdot {\tilde{C}}_{t}$ to obtain the new candidate value, which is measured by updating the value of each state.

(16) $$C_{t}=f_{t}\cdot C_{t-1}+i_{t}\cdot {\tilde{C}}_{t}$$

The fourth step is to finally determine the output. The output is based on the state of the neurons. The output gate of the sigmoid layer is used to determine which part of the neuron state is required to be output. The neuron state is then passed through the tanh layer (normalizing the output value to −1~1) and multiplied by the output of the sigmoid threshold to determine the required output.

(17) $$o_{t}=\sigma \left(W_{o}\cdot \left[h_{t-1},x_{t}\right]+b_{o}\right)$$

(18) $$h_{t}=o_{t}\cdot \tanh\left(C_{t}\right)$$

The LSTM can complete excellent learning and training in long-sequence scenes by virtue of its memory of sequences.

### 3.4. Prediction Model Based on LSTM

The velocity errors can be treated as time series data when the odometer works well, while the velocity and velocity errors change greatly when the odometer slips. When the odometer is working properly, the odometer provides the velocity as a reference to correct the IMU velocity, and the velocity is stored as a database that is used to build a training model based on LSTM to predict the velocity errors when odometer slips.

**Figure 6:**
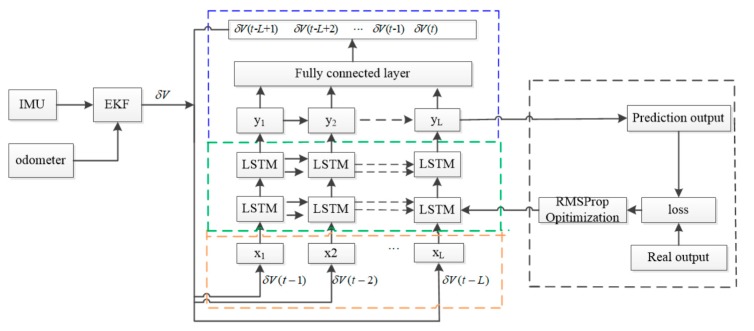
Training process.

With the velocity errors of the past n moments as the input and the velocity error information of the current time as the output, a model is developed using an LSTM network when the odometer slips. The overall framework of the network model and the training process are shown in Figure 6. V and δV are the velocity and velocity errors, respectively. δV(t) is the velocity error at the current time, δV(t−n)(n=1,2,…L,…) are the past velocity errors.

**Figure 7:**
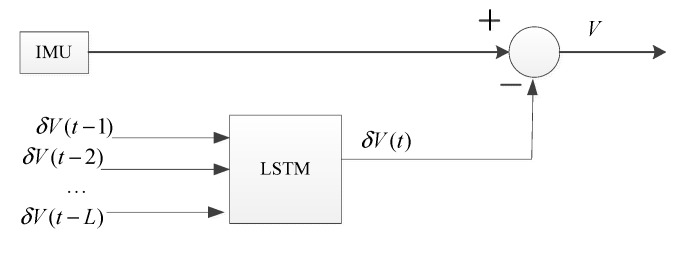
Prediction process.

Once the odometer slips, the velocity error can be obtained through prediction with the input velocity errors of the previous moments and the training model with an LSTM network. The prediction process when the odometer slips is shown in Figure 7.

For the LSTM network to predict the velocity error at the first time point, it is necessary to input the velocity error of the first L points as input, which is called the sequence length. Assuming that the velocity error data is $\Phi =\{\begin{array}{cccc} \delta V\left(t-1\right), & \delta V\left(t-2\right), & \cdots , & \delta V\left(t-L\right) \end{array}\}$, data preprocessing is needed. A single sample is a 1×L vector, the data set Φ is sample processed, (n−L)×L sample sets $\Psi =\{\psi_{1},\psi_{2},\ldots ,\psi_{n-L}\}$ are obtained. In which $\psi_{i}=\{\delta V\left(t-i\right),\delta V\left(t-i-1\right),\ldots ,\delta V\left(t-i-L\right)\},1\ll i\ll n-L,i\in N$.

After processing the original data, the time dimension of the sample expands, and the time dimension of the sample changes from one dimension to L dimensions. The sample set is divided into a training set $\Psi_{train}=\{\psi_{1},\psi_{2,}\ldots ,\psi_{m}\}$ and a test set $\Psi_{test}=\{\psi_{m+1},\psi_{m+2},\ldots ,\psi_{n-L}\}$, m<n−L,n∈N. In order to accelerate the training speed and prediction accuracy, it is necessary to normalize the samples in the training set $\Psi_{train}$. Z-score is used as a normalization method and the normalized training set is expressed as $\Psi_{train}^{\prime }=\{\psi_{1}^{\prime },\psi_{2}^{\prime },\ldots ,\psi_{m}^{\prime }\}$.

The model includes four modules: Input layer, hidden layer, output layer, and network training. Processed data as input layer is entered in the LSTM network. The hidden layer module is composed of the LSTM network and the full connection layer. The computational process in LSTM is described in the previous section. After calculating in the LSTM network layer, the output of the hidden layer is expressed as $y_{L}=\{y_{1},y_{2},\ldots ,y_{L}\}$, then through a full connection layer, the activate function chooses a linear activation function and finally outputs the one-dimensional velocity error data to be predicted.

Because of the large number of training samples, the mini-batch gradient descent algorithm is used to optimize the training process. Compared with the batch gradient descent, the small batch gradient descent selects one batch size sample each time to update the parameters, which saves the operation cost and improves the operation speed. The selection of learning rate has an important impact on the performance of the model and is often the most difficult parameter to debug. In order to reduce the difficulty of parameter debugging and optimize the performance of the model, the hyperparametric optimization algorithm is used to optimize the learning rate. The commonly used adaptive learning rate optimization algorithms are Adaptive Gradient Algorithm (AdaGrad), Root Mean Square Prop(RMSProp), Adaptive Moment Estimation(Adam), etc. RMSProp algorithm is chosen as the model super parametric optimization algorithm. The specific parameters for network training are as follows, the batch size is set as 1000, and the step is 15. Activation function is Rectified Linear Units(RELU). Number of hidden nodes is fifteen. The learning rate is set as 0.05.

## 4. Experiments and Results

### 4.1. Data Set

**Figure 8:**
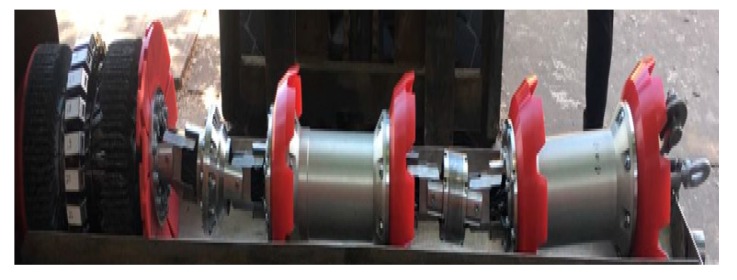
Centerline measurement system in field test.

The field test of the centerline measurement was carried out for a crude oil pipeline of a company. Figure 8 shows the centerline measurement used in the experiment. During the entire test process, the inspector ran in the pipeline about 157 km at an average speed of 1.16 m/s and a maximum speed of 1.27 m/s. The sampling rate of pipeline centerline measurement was 200 Hz. A ground marker box which provides GPS information was set every 2 km directly above the centerline of the buried pipeline to obtain the time at which the inspector system passed through the AGM box, in order to correct the error of measurement results. Using the coordinate calculation algorithm, the data of the IMU, odometer, and GPS marker on the ground were calculated to obtain more accurate position information of the pipeline centerline.

The coordinates of each point at the pipeline centerline can be obtained through the centerline calculation, and the amount of data is very large. Girth weld of the pipeline is the main feature of the pipeline, and the position of the weld can be obtained in engineering. After the girth weld recognition, the data at the girth weld were taken as the data set. The number of training samples was 12,000, and the number of test samples was 1500.

### 4.2. Weld Identification

**Figure 9:**
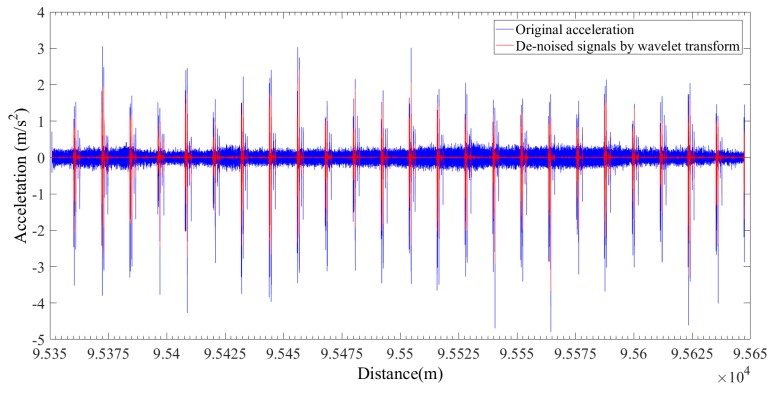
Comparison between original signals of inertial measurement acceleration and de-noised signals through wavelet transform.

**Figure 10:**
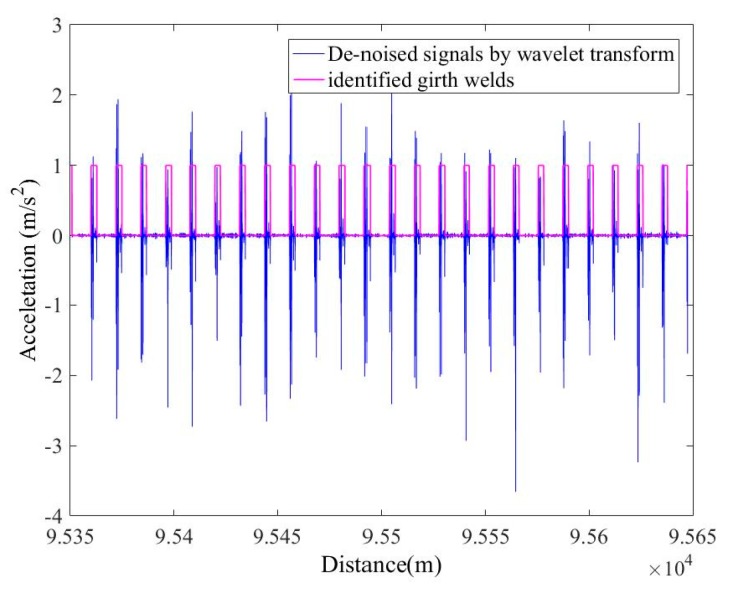
Identification of pipeline girth weld.

**Figure 11:**
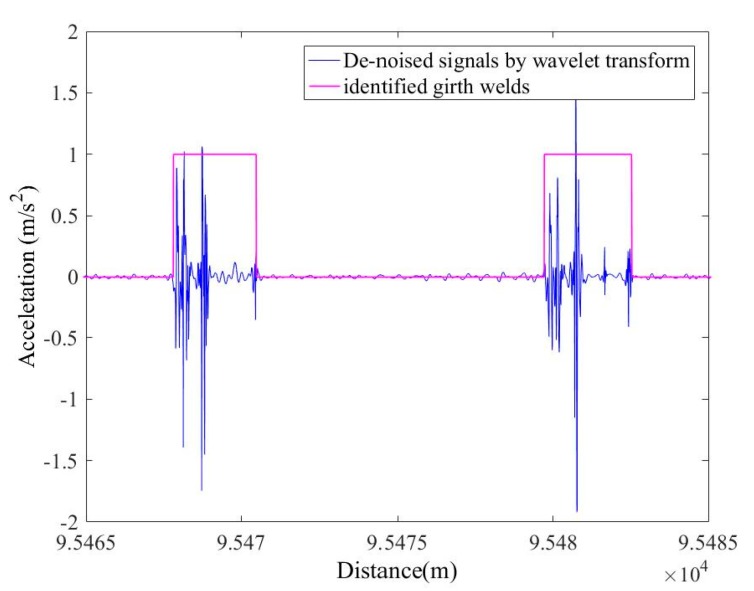
Zoom-in of identification of pipeline girth welds.

The output data of the accelerometer when the in-line inspection tool runs in the pipeline and the de-noised signals through the WT are shown in Figure 9. The de-noised accelerometer signals can be effectively used for the identification of the girth weld of the pipeline, and the result obtained is shown in Figure 10 and Figure 11. In order to ensure the correctness of the identification, the distance and time of the odometer passing through the girth weld were reviewed. As shown in Figure 11, the axial distance of the pink square is the length of the PIG. After measuring, the length was equal to the actual length of the PIG, which proves the validity and accuracy of the method. Therefore, each weld information can be identified by the acceleration signal after wavelet de-noising. With the collected IMU data, the multi-sensor data fusion calculation was carried out and the centerline ordinates were obtained. According to the centerline ordinates and the identified girth welds, the pipe length of each section can be calculated, and compared with the actual engineering data.

### 4.3. Test Results

After the field test, the geocoordinates were solved for the whole pipeline. After the welds were identified, the pipe length calculation was performed according to the identified welds and compared with the actual pipeline. The absolute error between the measurement point and the actual was used to evaluate the method. It was found that most of the errors between the measured centerlines and actual pipeline met the requirement in engineering. However, some centerlines of measurement deviated from the actual pipeline due to the odometer slip, this required post-processing correction by the proposed method.

**Figure 12:**
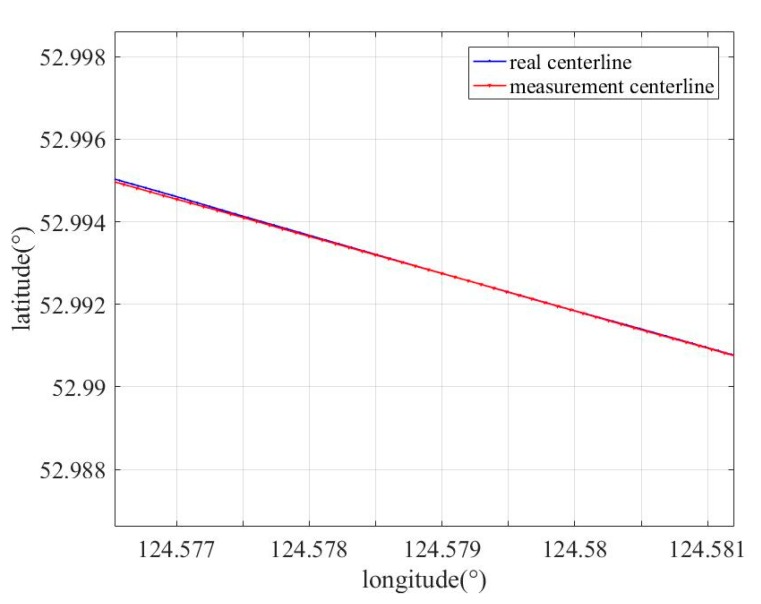
Measured length and actual length comparisons of pipeline.

Figure 12 shows a comparison of the measured length and actual length of a pipeline, which was consistent with the actual length. Table 1 shows the error between the measured and actual values at some measuring points. It can be observed from Figure 12 and Table 1 that the error of the measured and actual positions obtained by the calculation method was less than 3 m, which meets the requirements of practical engineering.

**Figure 13:**
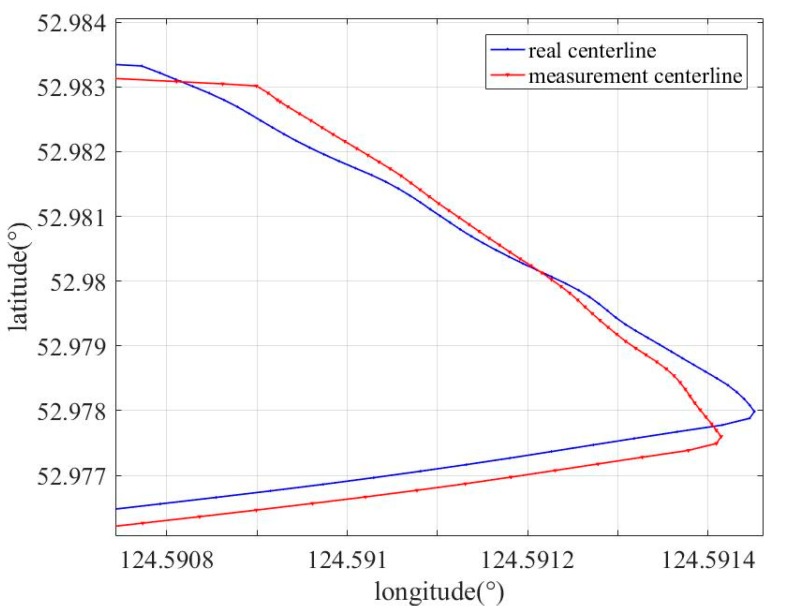
Measured length and actual length comparisons of pipeline at corners.

Figure 13 shows the position comparison of a section of the pipeline, in which the pipe length after the weld identification was not consistent with the actual pipe length. Table 2 lists the error between the measured and actual values of some measuring points when the odometer slips. It can be observed from Figure 13 and Table 2 that there is a large deviation between the measured centerline and the actual position of the pipeline at the bend. Under the same experimental conditions, the ground marking systems placed at each kilometer were activated, the abnormal data shows that the odometer slips at the bend, and the velocity reference information provided was inaccurate. At this time, the velocity cannot be used to correct the track of the IMU. In this paper, a compensation method of combined EKF and girth weld identification based on WT with LSTM network is proposed, and the model is established using the velocity error information when no slip occurs, to predict the velocity when the odometer slips for further assisting the coordinate measurement based on MEMS-IMU.

**Figure 14:**
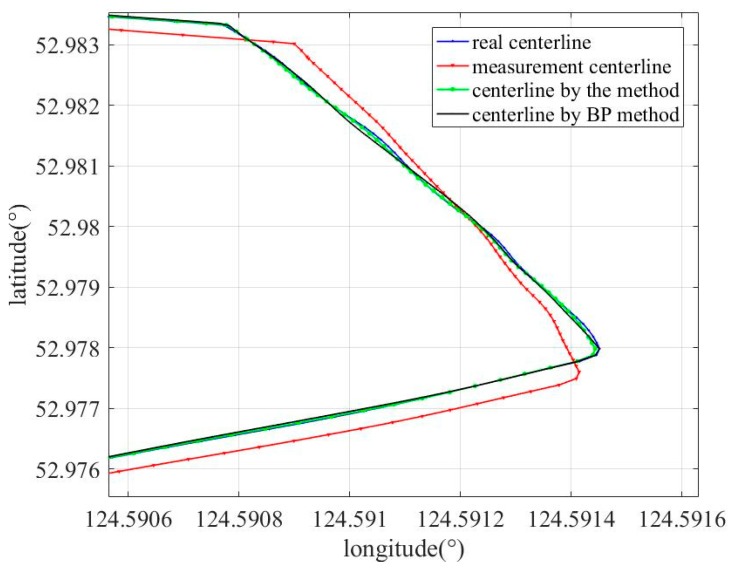
Centerline modified by different methods.

**Figure 15:**
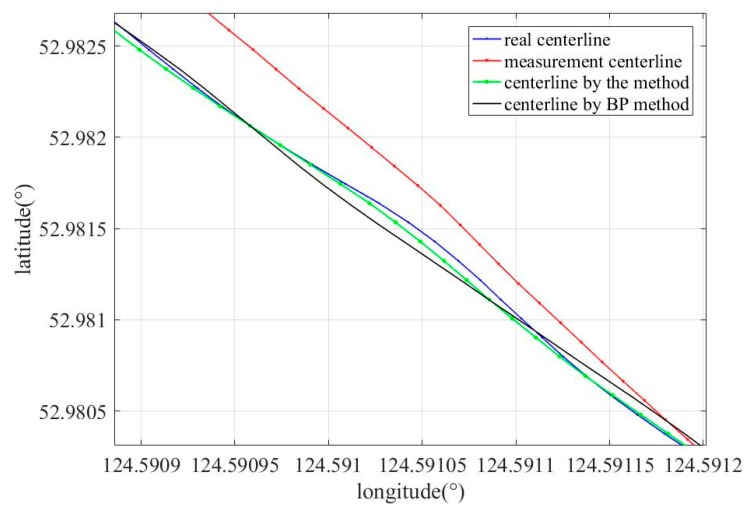
Zoom-in of centerline modified by different methods.

In order to verify the effectiveness of the proposed method, a method in which a BP neural network was used to replace the LSTM network (BP method) was implemented, and the results of the method were compared with the results of the used BP method. In the BP method, a BP neural network with a three-layer structure was used, and the activation functions were sigmoid. Figure 14 and Figure 15 show the comparison results between the results of the different algorithms; the measurement centerline, real trajectory, centerline corrected by the proposed method, and centerline corrected by the BP method are indicated by the red, blue, green, and black curves, respectively. As shown in Figure 15, the corrected centerline obtained by the proposed method is close to the real trajectories, and the corrected centerline obtained by BP method exhibits larger deviation from the real centerline. Table 3 shows the errors between the measured centerline obtained on using different methods and the actual centerline at some points when the odometer slips. Error 1, Error 2, and Error 3 represent the error between corrected centerline by the proposed method and the real centerlines at some points, the error between corrected centerlines by the BP method and the real centerline at some points, and the error between measured centerlines and real centerlines at some points, respectively. From Figure 15 and Table 3, it can be clearly observed that the performance of the proposed method is superior to the BP method.

**Figure 16:**
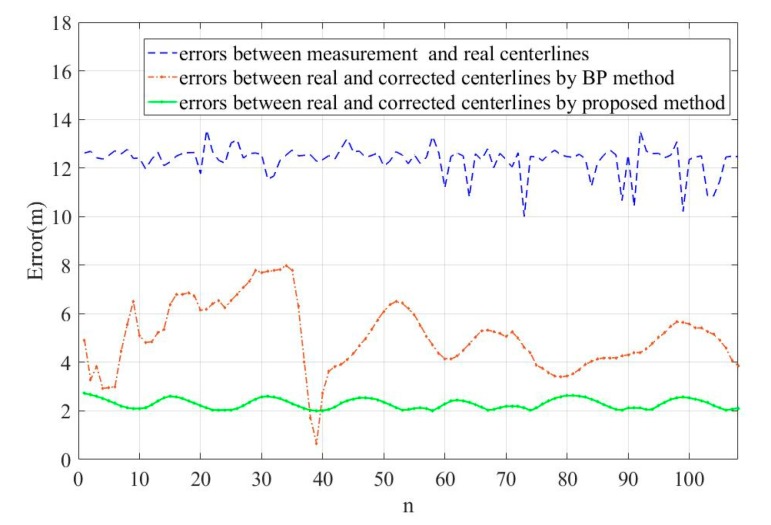
Error results between different algorithms.

Figure 16 shows the absolute position error results obtained for different algorithms. It can be seen that the error between real and corrected centerlines by the proposed method is the smallest and the corrected pipeline trajectory obtain by the proposed method is the closest approach to the real pipeline trajectory.

Table 4 shows the mean of the absolute position error (MAE) results obtained for different algorithms. The error between measured and real centerlines was 8.75 m, and the error between real and corrected centerlines by the BP method was 4.84 m. Based on the calculations, it can be observed that the mean of absolute position errors between real and corrected centerlines by the proposed method was 2.02 m, which is less than the error between real and corrected centerlines by the BP method, this is a reduction of approximately 58%. Therefore, it can be concluded that the proposed method can provide a better performance in terms of positioning accuracy.

## 5. Conclusions

In this paper we propose an error compensation method for pipeline centerline measurement during odometer slips to improve the measurement and positioning precision. Firstly, the pipeline centerline and coordinates measurement are realized based on multi-sensor fusion method with MEMS-IMU, odometer, and ground marker. Then, girth weld identification based on the wavelet transform is used to identify whether each length of pipeline segment is equal to the manufactured pipeline segment. Then an error compensation method based on the LSTM model is put forward to correct the errors due to odometer slip. When the odometer works well, the system completes the pipeline centerline measurement and stores the required data for training the model based on LSTM network; once the odometer slips, the training model is used to predict the odometer velocity error for coordinate calculation. The method utilizes a large amount of data from the whole pipeline, effectively establishing a model to predict the speed errors for correcting the MEMS-IMU, which provides an approach when the odometer slips. The field experiments show that the proposed method can effectively measure the coordinates and centerline of pipelines and the measurement errors reduced by 58% compared to the other method. The proposed method provides a solution for error compensation during odometer slips and can be used to realize high-precision measurements.

## Figures and Tables

**Table 1:** Error between measured and actual points.

No.	Measured Longitude (°)	Measured Latitude (°)	Real Longitude (°)	Real Latitude (°)	Error (m)
1	124.57948420	57.99230740	124.57949540	52.99230316	0.89
2	124.57957860	52.99222114	124.57959320	52.99221432	1.24
3	124.57967490	52.99213328	124.57969110	52.99212434	1.47
4	124.57977180	52.99204554	124.57978930	52.99203450	1.70
5	124.57986900	52.99195810	124.57988700	52.99194583	1.82
6	124.57996600	52.99187037	124.57998650	52.99185644	2.07
7	124.58006130	52.99178334	124.58008640	52.99176721	2.46

**Table 2:** Error between measured point and actual point when odometer slips.

No.	Measured Longitude (°)	Measured Latitude (°)	Real Longitude (°)	Real Latitude (°)	Error (m)
1	124.59086180	52.98304654	124.59082880	52.98300868	4.8
2	124.59089940	52.98301530	124.59084690	52.98290333	12.9
3	124.59091210	52.98290655	124.59086330	52.98279767	12.5
4	124.59092530	52.98277223	124.59089130	52.98258690	20.7
5	124.59093400	52.98269515	124.59090410	52.98247967	24.1
6	124.59095960	52.98248108	124.59092990	52.98227001	23.6
7	124.59099720	52.98215879	124.59097420	52.98195708	22.5

**Table 3:** Errors between the measured centerline obtained by different methods and the actual centerline.

No.	Corrected Longitude (°)/Latitude (°) by the Method	Corrected Latitude (°)/Latitude (°) by BP Method	Measured Latitude (°)/Latitude	Real Longitude (°)/Latitude (°)	Error1 (m)	Error2 (m)	Error3 (m)
1	124.59105660/52.98142933	124.59107020/59.98152002	124.59105650/52.95131436	124.5910489/52.98142929	1.37	7.20	8.74
2	124.59110290/52.98100672	124.59111240/52.98109288	124.59111630/52.98089624	124.59109800/52.98100684	1.23	6.80	8.74
3	124.59126830/52.97975655	124.59124630/52.97981705	124.59127370/52.97964474	124.59126130/52.97975669	1.33	4.80	8.83
4	124.59140960/52.97849841	124.59136200/52.97854017	124.59141090/52.97838565	124.59140460/52.97849879	1.23	3.80	8.90
5	124.59140960/52.97849841	124.59136200/52.97854017	124.59141090/52.97838565	124.59140460/52.97849879	1.23	3.80	8.90
6	124.59127270/52.97747027	124.59140920/52.97749228	124.59122280/52.97736086	124.59127180/52.97746965	1.60	6.76	8.87
7	124.59102940/52.97696379	124.59118140/52.97697250	124.59094730/52.97685904	124.59102500/52.97696405	1.20	7.45	9.04
8	124.59022870/52.97556484	124.59034110/52.97555870	124.59015520/52.97546116	124.59022100/52.97556578	1.37	5.70	8.80

**Table 4:** Mean of absolute position error.

Error (m)	Error between Measured and Real Centerlines (m)	Error between Real and Corrected Centerlines by the BP Method (m)	Error between Real and Corrected Centerlines by the Proposed Method (m)
The mean	8.75	4.84	2.02
